# Real-world efficacy and safety of Tenecteplase versus Alteplase in acute ischemic stroke: a propensity score-matched analysis

**DOI:** 10.3389/fneur.2026.1826373

**Published:** 2026-05-12

**Authors:** Peng Zhang, Liangyun Sun, Liangxue Wang

**Affiliations:** Department of Neurology, People's Hospital of Anji, Huzhou, China

**Keywords:** acute ischemic stroke, propensity score matching, real-world study, reperfusion therapy, Tenecteplase

## Abstract

**Background:**

Real-world evidence comparing Tenecteplase and Alteplase for acute ischemic stroke (AIS) remains limited. This study aimed to evaluate the comparative efficacy and safety of these thrombolytic agents in an unselected patient cohort.

**Methods:**

This single-center retrospective study analyzed AIS patients undergoing reperfusion therapy. Propensity score matching (PSM) was employed to balance baseline covariates between Tenecteplase and Alteplase groups. The primary outcome was functional independence (modified Rankin Scale 0–2) at 90 days. Safety endpoints included symptomatic intracranial hemorrhage (sICH) and mortality.

**Results:**

Among 371 eligible patients, 68 (18.3%) received Tenecteplase. In the matched cohort, Tenecteplase demonstrated comparable efficacy to Alteplase regarding functional independence (*p* > 0.05). Although the incidence of sICH was numerically higher in the Tenecteplase group, the difference was not statistically significant (*p* = 0.449). Workflow efficiency, measured by door-to-needle time, was similar between groups.

**Conclusion:**

Tenecteplase exhibits a non-inferior efficacy and safety profile compared to Alteplase in a real-world setting characterized by high baseline stroke severity. These findings support Tenecteplase as a practical and effective therapeutic alternative for routine AIS management.

## 1 Introduction

Acute ischemic stroke (AIS) remains a leading cause of mortality and long-term disability globally (1, 2). Hyperacute management represents a combination of intravenous thrombolysis (IVT) and endovascular thrombectomy (EVT), based on the timely restoration of cerebral blood flow by means of reperfusion therapy (3, 4). Alteplase is a recombinant tissue plasminogen activator (rt-PA) that has been used as the standard thrombolytic activity over decades (5, 6). Nonetheless, its clinical use is limited by a short half-life requiring a continuous one-hour infusion following an initial bolus, which could frustrate logistical operations, especially during inter-hospital transfers or the transition to endovascular procedures (7, 8).

A genetically engineered derivative of Alteplase, Tenecteplase, has pharmacokinetic properties that offer specific benefits: it has a longer half-life, is more fibrin-specific, and exhibits greater resistance to plasminogen activator inhibitor-1 (9, 10). The characteristics of these properties also permit administration by a single intravenous bolus, which theoretically simplifies administration and might enhance reperfusion (11). After the seminal results of randomized controlled trials (RCTs) including EXTEND-IA TNK and AcT that showed the non-inferiority, and in certain subsets of large vessel occlusion (LVO), the superiority of Tenecteplase over Alteplase has been demonstrated, more guidelines have endorsed its use (12, 13).

Even the persuasive nature of the RCTs notwithstanding, the extrapolation of the results to the routine clinical practice deserves critical examination (14). Clinical trials are usually conducted under very strict inclusion criteria, which often exclude older people, individuals with severe comorbid conditions, or those with severe neurological deficits thus limiting their representativeness of other individuals or groups in generalized real-world settings (15). Moreover, the information on the safety and effectiveness of Tenecteplase compared to other county-level stroke centers, where workflow processes and resources are not comparable to those of a comprehensive academic center, is also rather limited (16).

This knowledge gap prompted this study to evaluate the comparative efficacy and safety of Tenecteplase versus Alteplase in patients with AIS outside tertiary care in a county-level stroke center. We specifically evaluated functional outcomes, workflow efficiency measures (door-to-needle time) and safety profiles, such as symptomatic intracranial hemorrhage. Considering the inherent bias of observational data, propensity score matching (PSM) was employed to correct the baseline confounders to offer a strong evaluation of the performance of Tenecteplase in a mixed, unselected patient group.

## 2 Materials and methods

### 2.1 Study design and ethical considerations

This single-center, retrospective observational study was conducted at People’s Hospital of Anji. The study protocol underwent expedited review and was approved by the Medical Ethics Committee of People’s Hospital of Anji (Approval Number: 20250047-1). Given the retrospective nature of the research, which involved the analysis of de-identified medical records and posed minimal risk to participants, the requirement for individual informed consent was waived by the institutional review board. All patient data were anonymized prior to analysis to ensure confidentiality, and the study was conducted in strict adherence to the ethical principles outlined in the Declaration of Helsinki.

### 2.2 Patient selection

We screened the electronic medical records of consecutive adult patients (aged ≥18 years) admitted with a primary diagnosis of acute ischemic stroke (AIS) who underwent reperfusion therapy between January 2024 and December 2025. Inclusion criteria were: (1) clinical diagnosis of AIS confirmed by neuroimaging (computed tomography [CT] or magnetic resonance imaging [MRI]); (2) treatment with intravenous thrombolysis (IVT) using either Alteplase or Tenecteplase, with or without subsequent endovascular thrombectomy (EVT); and (3) availability of complete baseline and follow-up data. Patients were excluded if they had: (1) a final diagnosis mimicking stroke; (2) incomplete documentation regarding thrombolytic agents or key outcome measures; or (3) pre-stroke disability (modified Rankin Scale [mRS] > 2).

### 2.3 Treatment protocols and data collection

Reperfusion therapies were administered in accordance with the prevailing guidelines at the time of treatment. The patients under the Alteplase group had a standard dosage of 0.9 mg/kg (maximum dosage of 90 mg) of which 10% was administered as an initial bolus and the remainder was infused over 60 min. Patients in the Tenecteplase group received a single intravenous bolus of 0.25 mg/kg (maximum 25 mg) of recombinant human Tenecteplase (rhTNK-tPA, Guangzhou Recomgen Biotech Co., Ltd., China). The selection of the thrombolytic agent was determined by the treating physician based on clinical judgment and drug availability. In patients with large vessel occlusions (LVO), bridging therapy or direct mechanical thrombectomy was performed using stent retrievers, aspiration, or the combined technique.

The hospital information system was searched to extract baseline demographic, clinical, laboratory, and imaging data. Major workflow indicators, such as the onset-to-door time (ODT) and door-to-needle time (DNT) were documented. The severity of stroke was measured with the National Institutes of Health Stroke Scale (NIHSS) at admission, after 24 h and discharge.

### 2.4 Outcome measures

The primary efficacy outcome was functional independence at 90 days, defined as an mRS score of 0 to 2. Secondary efficacy outcomes included excellent functional outcome (mRS 0–1) and early neurological improvement (ENI). Safety outcomes comprised symptomatic intracranial hemorrhage (sICH) and 90-day all-cause mortality. sICH was defined according to the European Cooperative Acute Stroke Study (ECASS) III criteria.

### 2.5 Statistical analysis

The continuous variables were measured and presented as mean ± standard deviation (SD) or median [interquartile range, IQR] and compared using the Student’s *t*-test or the Mann–Whitney *U*-test, as appropriate. Categorical variables were presented as frequencies (percentages) and compared using the Chi-square test or Fisher’s exact test. A complete-case analysis strategy was employed to handle missing data, and patients with missing critical variables were excluded from the multivariable models. As this was a retrospective observational study, a formal *a priori* sample size calculation was not performed; rather, the sample size was determined by the total number of consecutive eligible patients treated during the specified study period to maximize statistical power.

To reduce selection bias inherent in the observational design, a propensity score matching (PSM) analysis was performed without replacement using the MatchIt package in R. Balancing the covariates of the baseline was done using a 1:1 nearest-neighbor matching algorithm at a caliper width of 0.2 to achieve equilibrium between the Alteplase and the Tenecteplase groups. The model covariates included age, sex, baseline NIHSS, systolic blood pressure, onset-to-door time, use of endovascular thrombectomy, and history of hypertension, diabetes, and atrial fibrillation. Covariate balance was rigorously evaluated using absolute standardized mean differences (SMD), with an SMD < 0.1 indicating good balance, supplemented by the assessment of variance ratios for continuous variables. Multivariable logistic regression models were further employed to estimate adjusted odds ratios (aOR) for clinical outcomes. Prior to regression, multicollinearity among independent variables was assessed using the Variance Inflation Factor (VIF), with a VIF < 5 indicating no significant collinearity. The R software (Version 4.4.1) was used in all statistical analyses, in which a two-sided *p*-value less than 0.05 was deemed to be statistically significant.

## 3 Results

### 3.1 Study population dynamics and baseline clinical characteristics

**Figure 1:**
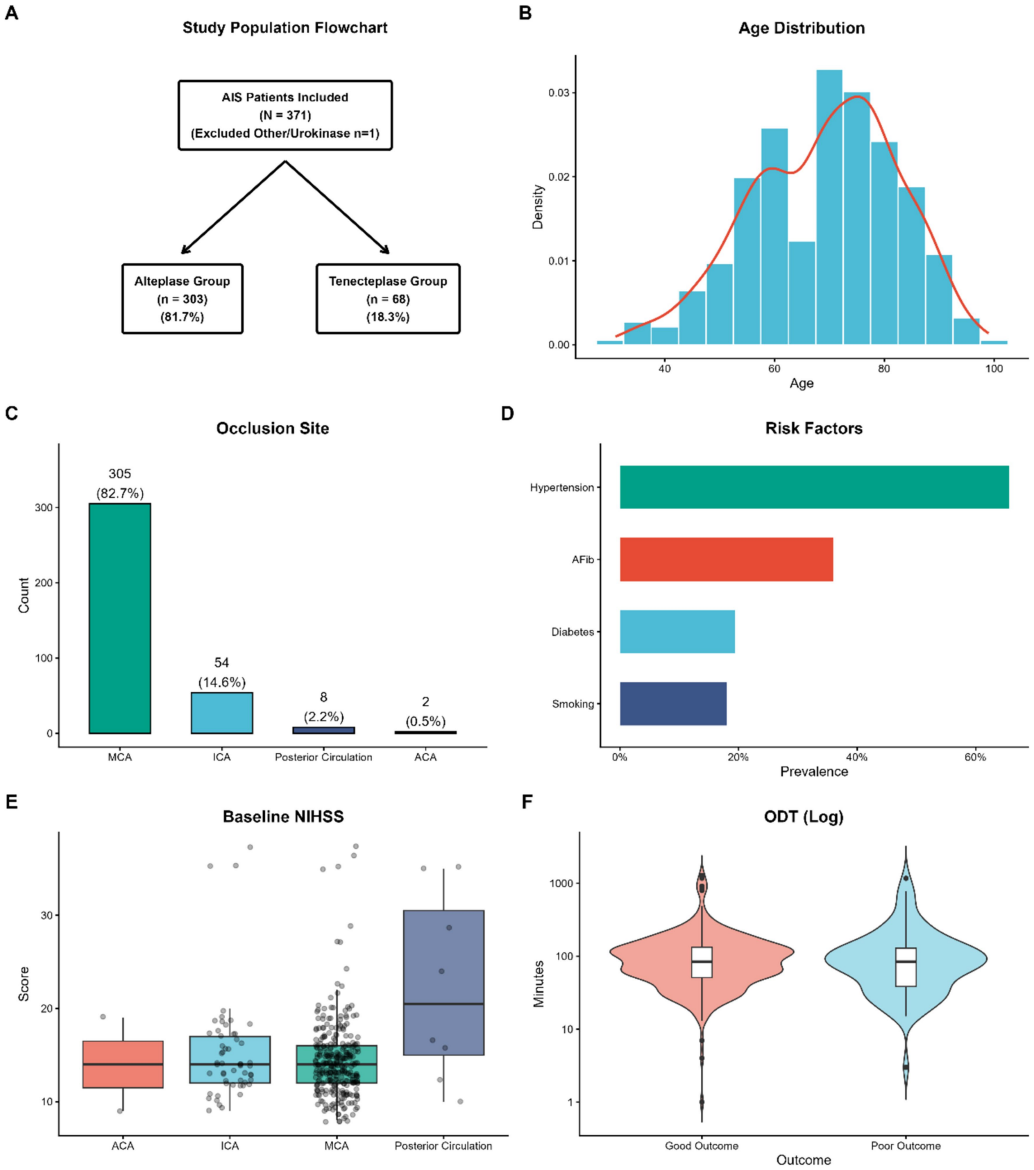
Study population dynamics and baseline clinical characteristics. **(A)** Flowchart illustrating the patient inclusion process and treatment stratification (IV thrombolysis only vs. endovascular/bridging therapy). **(B)** Histogram and density plot showing the age distribution of the cohort. **(C)** Distribution of arterial occlusion sites, with the middle cerebral artery (MCA) being the most prevalent. **(D)** Prevalence of major vascular risk factors; hypertension was the most common comorbidity. **(E)** Boxplots of baseline NIHSS scores stratified by occlusion site, highlighting higher severity in posterior circulation and ICA occlusions. **(F)** Violin plots of onset-to-door time (ODT) distribution (log scale) stratified by functional outcome. AIS, acute ischemic stroke; IV, intravenous; MCA, middle cerebral artery; ICA, internal carotid artery; ACA, anterior cerebral artery; NIHSS, National Institutes of Health Stroke Scale.

A total of 372 patients diagnosed with acute ischemic stroke (AIS) were initially identified for screening. The final analytic cohort comprised 371 patients following the exclusion of one patient treated with urokinase (Figure 1A). The study population was stratified into two treatment arms of treatment depending on the type of thrombolytic agent applied to them, the Alteplase group (*n* = 303, 81.7%), and the Tenecteplase group (*n* = 68, 18.3%).

Detailed baseline demographic and clinical characteristics are summarized in Table 1. The average ages of both the Alteplase and the Tenecteplase groups were similar (69.17 ± 13.06 vs. 68.90 ± 14.22 years; *p* = 0.878) and the age distributions were as shown in Figure 1B. However, a significant disparity in sex distribution was observed, with a higher proportion of males in the Alteplase group as opposed to the Tenecteplase group (65.0% vs. 47.1; *p* = 0.009). Concerning vascular risk factors hypertension was the most common comorbidity in the whole cohort, then atrial fibrillation, and diabetes mellitus (Figure 1D). While the prevalence of these major risk factors was balanced between the two groups, patients in the Alteplase group had a significantly higher prevalence of previous stroke or transitory ischemic attack (TIA) (15.5% vs. 4.4%; *p* = 0.026).

**Table 1:** Baseline characteristics of patients stratified by thrombolytic agent.

Variables	Alteplase (*n* = 303)	Tenecteplase (*n* = 68)	*P*-value
Demographics			
Age, years, mean ± SD	69.17 ± 13.06	68.90 ± 14.22	0.878
Male, *n* (%)	197 (65.0)	32 (47.1)	**0.009**
BMI, kg/m^2^, mean ± SD	22.95 ± 4.01	22.95 ± 3.90	0.989
Vascular risk factors, *n* (%)			
Hypertension	202 (66.7)	42 (61.8)	0.530
Diabetes mellitus	64 (21.1)	8 (11.8)	0.111
Atrial fibrillation	112 (37.0)	21 (30.9)	0.421
Previous stroke/TIA	47 (15.5)	3 (4.4)	**0.026**
Current smoking	55 (18.2)	12 (17.6)	1.000
Clinical characteristics on admission			
Baseline NIHSS, median [IQR]	14.00 [12.00, 16.00]	14.00 [12.00, 17.00]	0.280
ASPECTS, median [IQR]	9.00 [8.00, 10.00]	9.00 [8.00, 10.00]	0.471
Systolic BP, mmHg, mean ± SD	155.73 ± 19.54	152.93 ± 22.50	0.300
Diastolic BP, mmHg, mean ± SD	86.14 ± 12.62	84.28 ± 14.22	0.285
Occlusion site, *n* (%)			0.224
MCA (middle cerebral artery)	251 (82.8)	53 (77.9)	
ICA (internal carotid artery)	43 (14.2)	11 (16.2)	
Posterior circulation	6 (2.0)	2 (2.9)	
ACA/other	3 (1.0)	2 (2.9)	
Treatment and workflow			
Bridging therapy (IVT + EVT), *n* (%)	228 (75.2)	55 (80.9)	0.407
Onset-to-door time (ODT), min, median [IQR]	86.00 [53.50, 142.50]	73.50 [40.50, 110.00]	**0.028**
Door-to-needle time (DNT), min, median [IQR]	37.00 [31.00, 49.00]	36.00 [30.75, 45.25]	0.199

SD, standard deviation; IQR, interquartile range; BMI, body mass index; TIA, transient ischemic attack; NIHSS, National Institutes of Health Stroke Scale; ASPECTS, Alberta Stroke Program Early CT Score; BP, blood pressure; IVT, intravenous thrombolysis; EVT, endovascular thrombectomy.

Data are presented as *n* (%), mean ± SD, or median [IQR]. *p*-values < 0.05 are indicated in bold.

There were no significant differences in the neurological severity on admission, with the Alteplase and Tenecteplase median National Institutes of Health Stroke Scale (NIHSS) score of 14.00 (IQR [12.00–16.00]) and 14.00 (IQR [12.0017.00]) respectively (*p* = 0.280). Similarly, there were no significant differences in Alberta Stroke Program Early CT Score (ASPECTS) or the admission blood pressure profile. The Middle Cerebral Artery (MCA) was the primary occlusion site (82.8% in Figure 1C, Alteplase vs. 77.9% in Figure 1C, Internal Carotid Artery-IC) without any statistically significant difference in distribution (*p* = 0.224). Figure 1E also outlines the baseline NIHSS distribution by occlusion site with higher severity shown in patients with posterior circulation and ICA occlusion.

Regarding workflow measures, the Tenecteplase group had a much shorter Onset-to-Door Time (ODT) median than that of the Alteplase group (73.50 min [IQR 40.50–110.00] vs. 86.00 min [IQR 53.50–142.50]; *p* = 0.028), which is represented in the log-transformed distribution in Figure 1F. In contrast, there was no statistically significant difference in in-hospital processing efficiency, measured by Door-to-Needle Time (DNT) (37.00 min vs. 36.00 min; *p* = 0.199). The utilization of bridging therapy (intravenous thrombolysis and follow-up with endovascular thrombectomy) was 75.2% in the Alteplase group and 80.9% in the Tenecteplase group, showing no significant difference (*p* = 0.407).

### 3.2 Workflow efficiency metrics and their impact on outcomes

**Figure 2:**
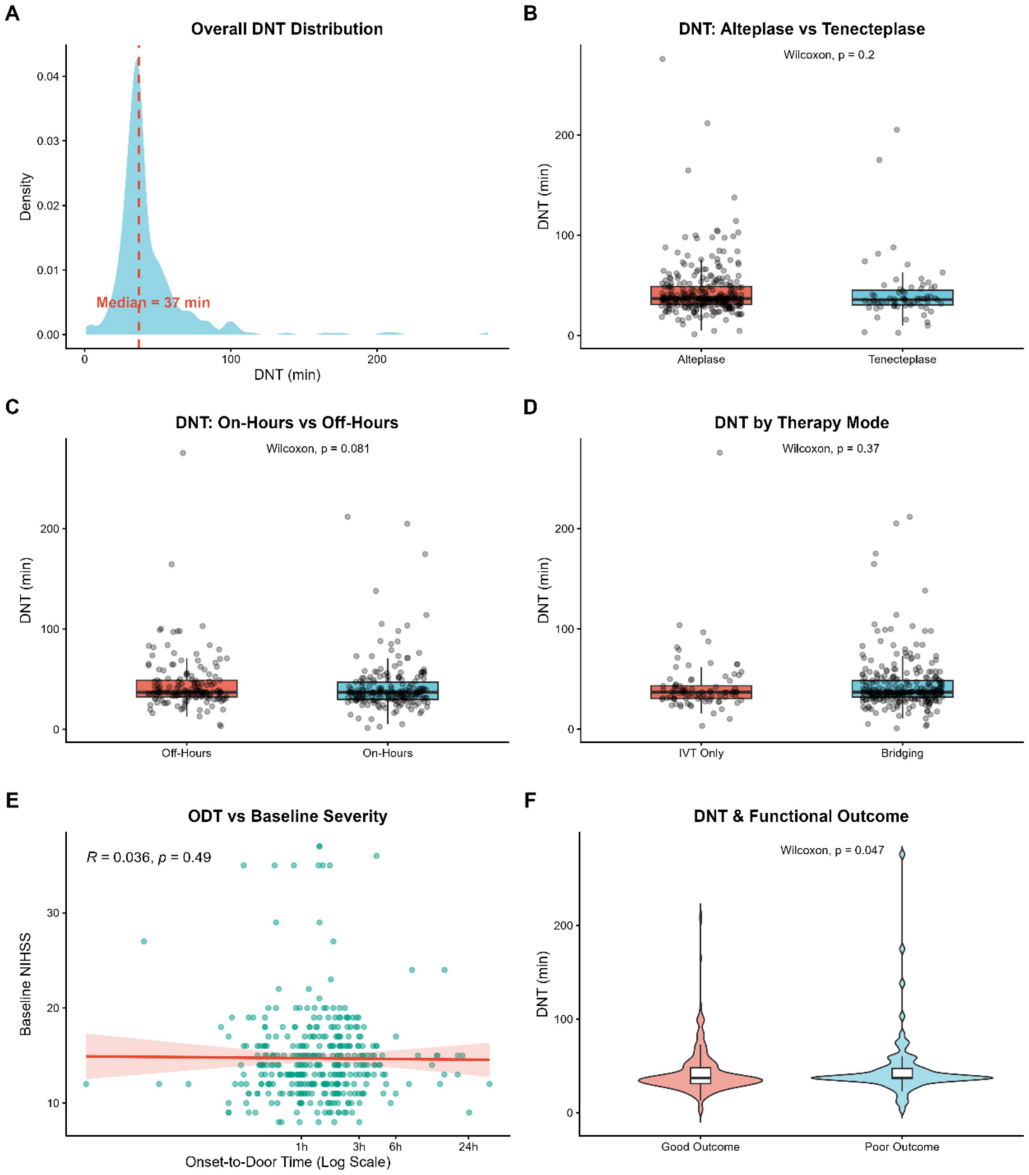
Workflow efficiency metrics and their impact on outcomes. **(A)** Density plot of the overall Door-to-Needle Time (DNT), with a median of 37 min. **(B)** Comparison of DNT between Alteplase and Tenecteplase groups (Wilcoxon test). **(C)** DNT comparison between working hours (On-Hours) and off-hours, showing no significant delay during off-hours. **(D)** DNT stratified by therapy mode (IVT Only vs. Bridging). **(E)** Correlation analysis between Onset-to-Door Time (ODT) and baseline NIHSS score (Spearman correlation). **(F)** Violin plot comparing DNT between patients with good (mRS 0–2) and poor outcomes, demonstrating significantly shorter DNT in the good outcome group. DNT, Door-to-Needle Time; ODT, Onset-to-Door Time; mRS, modified Rankin Scale.

Building upon the baseline characteristics which indicated a shorter pre-hospital delay (ODT) in the Tenecteplase group, we further analyzed in-hospital workflow metrics to assess the efficiency of the acute stroke pathway. The overall distribution of Door-to-Needle Time (DNT) for the entire cohort exhibited a median of 37.00 min (Figure 2A), reflecting a streamlined hyperacute management protocol.

In Figure 2B, a comparison analysis of DNT stratified on thrombolytic agent is provided. Despite the practical advantage of Tenecteplase requiring only a single bolus injection compared to the infusion required for Alteplase, the median DNT was not different between the two groups (Alteplase: 37.00 min [IQR 31.0049.00] vs. Tenecteplase: 36.00 min [IQR 30.7545.25]). The raw data distribution showed that there is no statistically significant difference (*p* > 0.05), suggesting that the choice of thrombolytic agent did not introduce variability into the speed of treatment initiation.

To assess the homogeneity of the quality of care, DNT was further subdivided into work shift and therapy mode. As shown in Figure 2C, treatment initiation during off-hours (median DNT 37.00 min) did not differ significantly from working hours (median DNT 37.00 min), which implies that the stroke team did not experience any shifts in performance levels with the time of presentation. Similarly, the complexity of decision-making involved in bridging therapy did not compromise the speed of thrombolysis. The median DNT in patients that underwent bridging therapy was statistically non-different to that of patients undergoing intravenous thrombolysis only (median 37.00 min), as shown in Figure 2D.

In addition, exploratory analyses were performed to study the association between the metrics of time and the clinical status. A scatter plot of ODT versus baseline NIHSS revealed no clear correlation (Figure 2E), implying that stroke severity did not inherently accelerate or delay hospital arrival in this cohort. However, regarding prognostic implications, patients achieving a good functional outcome had a distribution more concentrated toward shorter DNTs (mRS 0–2) as opposed to patients with poor functioning outcomes (Figure 2F), reinforcing the critical association between rapid reperfusion and favorable neurological recovery.

### 3.3 Reperfusion therapy modalities and technical outcomes

**Figure 3:**
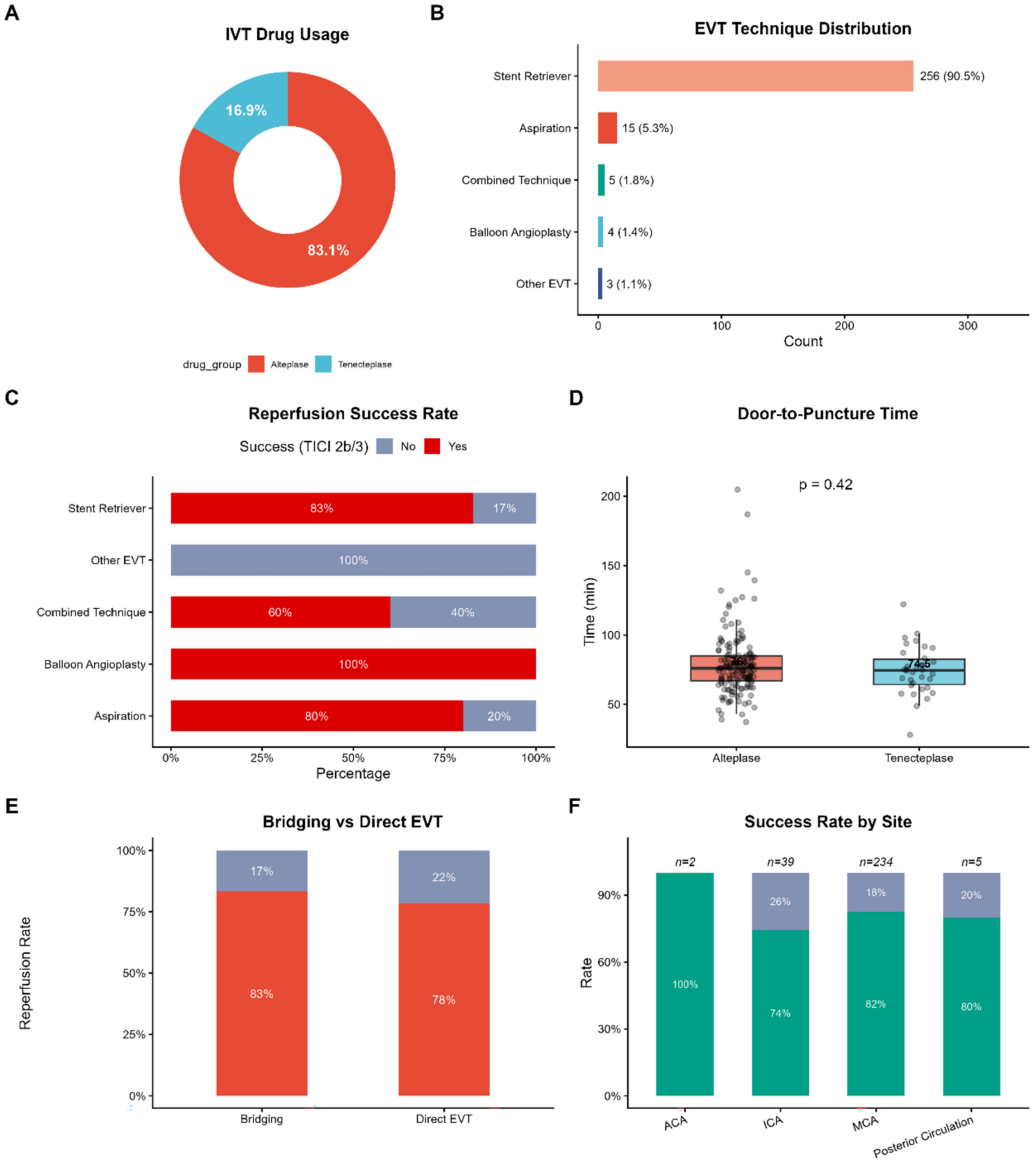
Reperfusion therapy modalities and technical outcomes. **(A)** Donut chart showing the proportion of thrombolytic agents used (Alteplase vs. Tenecteplase). **(B)** Distribution of endovascular treatment (EVT) techniques, with stent retrievers being the dominant modality. **(C)** Reperfusion success rates (mTICI 2b/3) stratified by EVT technique. **(D)** Door-to-puncture time (DPT) comparison between thrombolytic groups. **(E)** Comparison of reperfusion rates between bridging therapy and direct EVT. **(F)** Reperfusion success rates stratified by occlusion site; sample sizes are annotated above bars. IVT, intravenous thrombolysis; EVT, endovascular thrombectomy; mTICI, modified thrombolysis in cerebral infarction; DPT, door-to-puncture time.

Following the initiation of intravenous thrombolysis, the specific modalities of reperfusion therapy were analyzed to evaluate technical efficacy. Alteplase remained the standard of care for the majority of the cohort, accounting for 83.1% of cases, and Tenecteplase was applied in 16.9% (Figure 3A). The most widely used modality among patients who had undergone endovascular treatment (EVT) was the stent remover technique (90.5% of procedures), secondly was the aspiration procedure alone (5.3), and combined techniques (1.8%) (Figure 3B).

High rates of successful reperfusion (defined as mTICI 2b/3) were achieved across the major thrombectomy techniques. Specifically, the stent retriever group demonstrated a reperfusion success rate of approximately 83%, providing a robust benchmark for technical efficacy (Figure 3C). To determine the effect of the use of upstream thrombolytics on the mechanical procedure, we compared bridging therapy to direct mechanical thrombectomy. Figure 3E demonstrated that the successful reperfusion rate was 83% in the bridging therapy group compared with 78% in the direct EVT group. Although the rate of recanalization was found to be numerically higher in bridging therapy, both interventions had good technical results suggesting that the antecedent administration of lytics did not hinder the subsequent endovascular procedure.

As far as the efficiency of the intra-arterial workflow is concerned, Door-to-Puncture Time (DPT) was studied to determine if the choice of lytic agent affected the transition to groin puncture. Figure 3D showed that the median DPT in the Alteplase group was 76.00 min and the Tenecteplase group was 74.50 min. The lack of a statistically significant difference (*p* = 0.42) indicates that a transition between the emergency department to the angio-suite was smooth after either of the thrombolytic agents.

Lastly, we tested technical outcome upon basis of anatomical points of occlusion (Figure 3F). Although the habitual rates of successful reperfusion were mostly high regardless of the territories, the recanalization of the Internal Carotid Artery (ICA) was found to be numerically lower (74–80) than those of the Middle Cerebral Artery (MCA) (82–80) and Posterior Circulation (80–80) reflecting the inherent technical challenges associated with larger clot burdens or tandem lesions in proximal occlusions. The 100% success rate observed in the Anterior Cerebral Artery (ACA) group should be interpreted with caution due to the limited sample size (*n* = 2).

### 3.4 Safety outcomes and complication analysis

**Figure 4:**
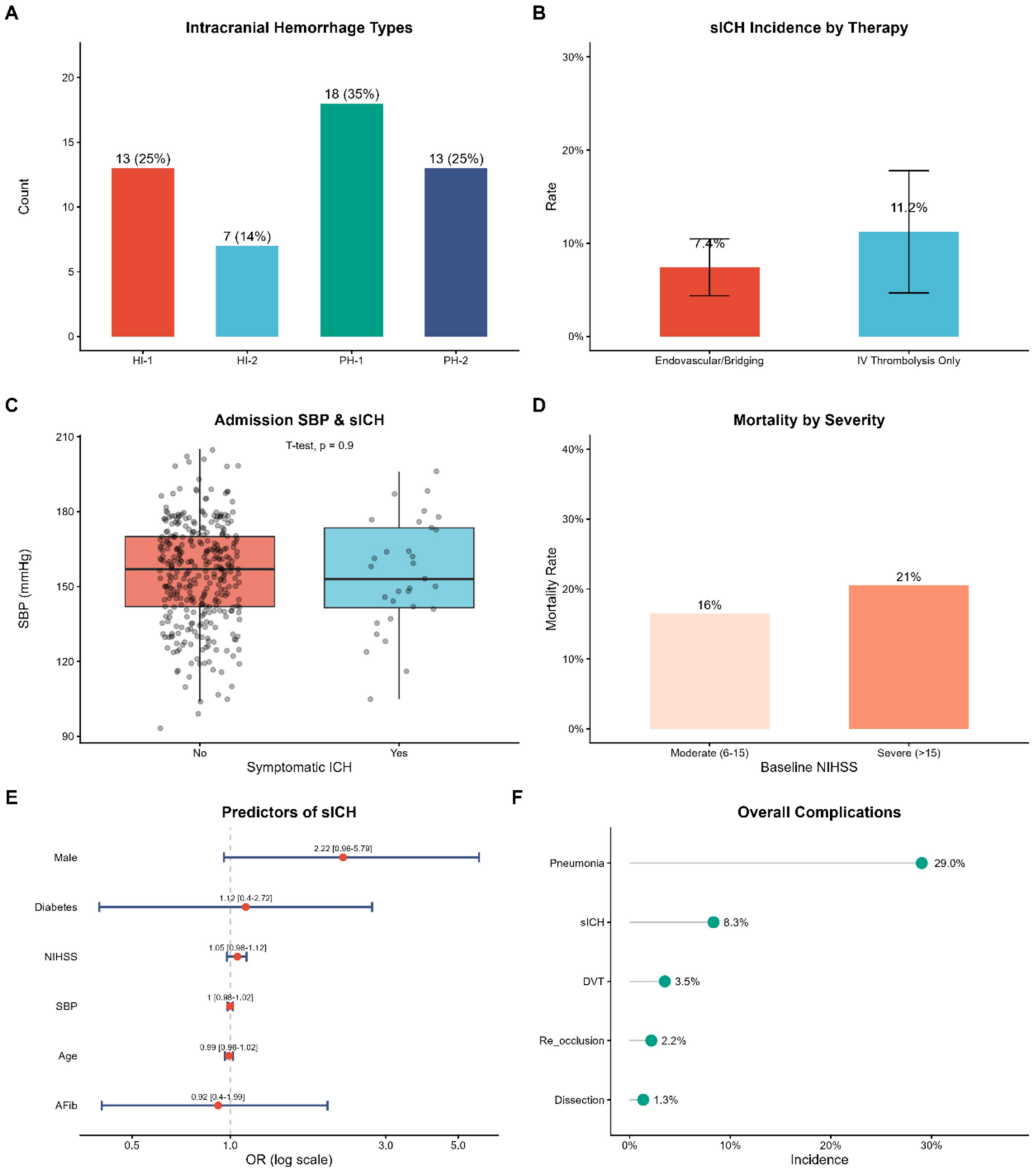
Safety outcomes and complication analysis. **(A)** Distribution of intracranial hemorrhage (ICH) subtypes according to the ECASS classification. **(B)** Incidence of symptomatic ICH (sICH) stratified by therapy mode. Error bars represent 95% confidence intervals. **(C)** Comparison of admission systolic blood pressure (SBP) between patients with and without sICH. **(D)** 90-day mortality rates stratified by baseline stroke severity (NIHSS). **(E)** Forest plot of multivariable logistic regression analysis identifying independent predictors of sICH. **(F)** Lollipop chart summarizing the incidence of overall complications. ICH, intracranial hemorrhage; sICH, symptomatic intracranial hemorrhage; HI, hemorrhagic infarction; PH, parenchymal hematoma; SBP, systolic blood pressure; DVT, deep vein thrombosis.

Safety remains a paramount concern in reperfusion therapy, particularly regarding the risk of intracranial hemorrhage (ICH). We characterized the spectrum of hemorrhagic transformation according to the ECASS classification. The most common subtypes as described in Figure 4A were parenchymal hematoma type 1 (PH-1) and hemorrhagic infarction type 1 (HI-1), while PH-2 accounted for 25% of all hemorrhagic events.

A comparative analysis of symptomatic intracranial hemorrhage (sICH) revealed a favorable safety profile for Tenecteplase. Table 2 shows that sICH was 8.8 percent and 8.3 percent in the Tenecteplase and the Alteplase groups respectively, with no statistically significant difference observed after multivariate adjustment (aOR 1.17, 95% CI 0.412.88; *p* = 0.742). In stratified data of therapeutic modality (Figure 4B) the incidence of sICH was 7.4% in endovascular/bridging group compared to 11.2% in IVT-only group. The overlapping confidence intervals suggest that the risk of hemorrhage in this real-world cohort was not disproportionately increased with the addition of mechanical thrombectomy.

**Table 2:** Comparative efficacy and safety outcomes of Tenecteplase versus Alteplase.

Outcomes	Alteplase (*n* = 303)	Tenecteplase (*n* = 68)	Unadjusted OR (95% CI)	Adjusted OR* (95% CI)	*P*-value
Efficacy outcomes					
Functional independence (mRS 0–2)	122 (40.3%)	26 (38.2%)	0.92 (0.53–1.57)	0.91 (0.52–1.58)	0.748
Excellent outcome (mRS 0–1)	81 (26.7%)	13 (19.1%)	0.65 (0.32–1.22)	0.65 (0.32–1.23)	0.202
Safety outcomes					
Symptomatic ICH	25 (8.3%)	6 (8.8%)	1.08 (0.39–2.58)	1.17 (0.41–2.88)	0.742
Mortality (90-day)	50 (16.5%)	15 (22.1%)	1.42 (0.73–2.69)	1.31 (0.65–2.50)	0.431

OR, odds ratio; CI, confidence interval; mRS, modified Rankin Scale; ICH, intracranial hemorrhage.

Data are presented as *n* (%).

*Adjusted for age, sex, baseline NIHSS score, history of hypertension, and onset-to-door time (ODT).

In order to investigate the possible predisposing factors to sICH, we examined the admission hemodynamics in addition to other clinical variables. Interestingly, admission systolic blood pressure (SBP) did not differ significantly between patients with sICH and without it (*p* = 0.9, Figure 4C), which could also indicate that our center implemented strict protocols related to the management of blood pressure during the admission period. Consistent with this, the multivariable logistic regression analysis (Figure 4E) indicated that neither SBP nor baseline NIHSS were independent predictors of sICH in this particular sample, although male sex showed a trend toward increased risk (OR 2.22, 95% CI 0.96–5.79) without reaching statistical significance.

On the overall survival and systemic complications, the 90-day mortality rate showed a strong correlation with the baseline stroke severity and increased with the stroke severity to 16 percent in patients with moderate stroke and 21 percent in patients with severe stroke (Figure 4D). Importantly, the choice of thrombolytic agent did not influence mortality (16.5 vs. 22.1; Table 2). In addition to neurological events, pneumonia was also the most common systemic complication (29.0% of the study population), followed by sICH (8.3) and deep vein thrombosis (3.5) (Figure 4F), highlighting the need for comprehensive post-stroke care.

### 3.5 Clinical efficacy outcomes in the entire cohort

**Figure 5:**
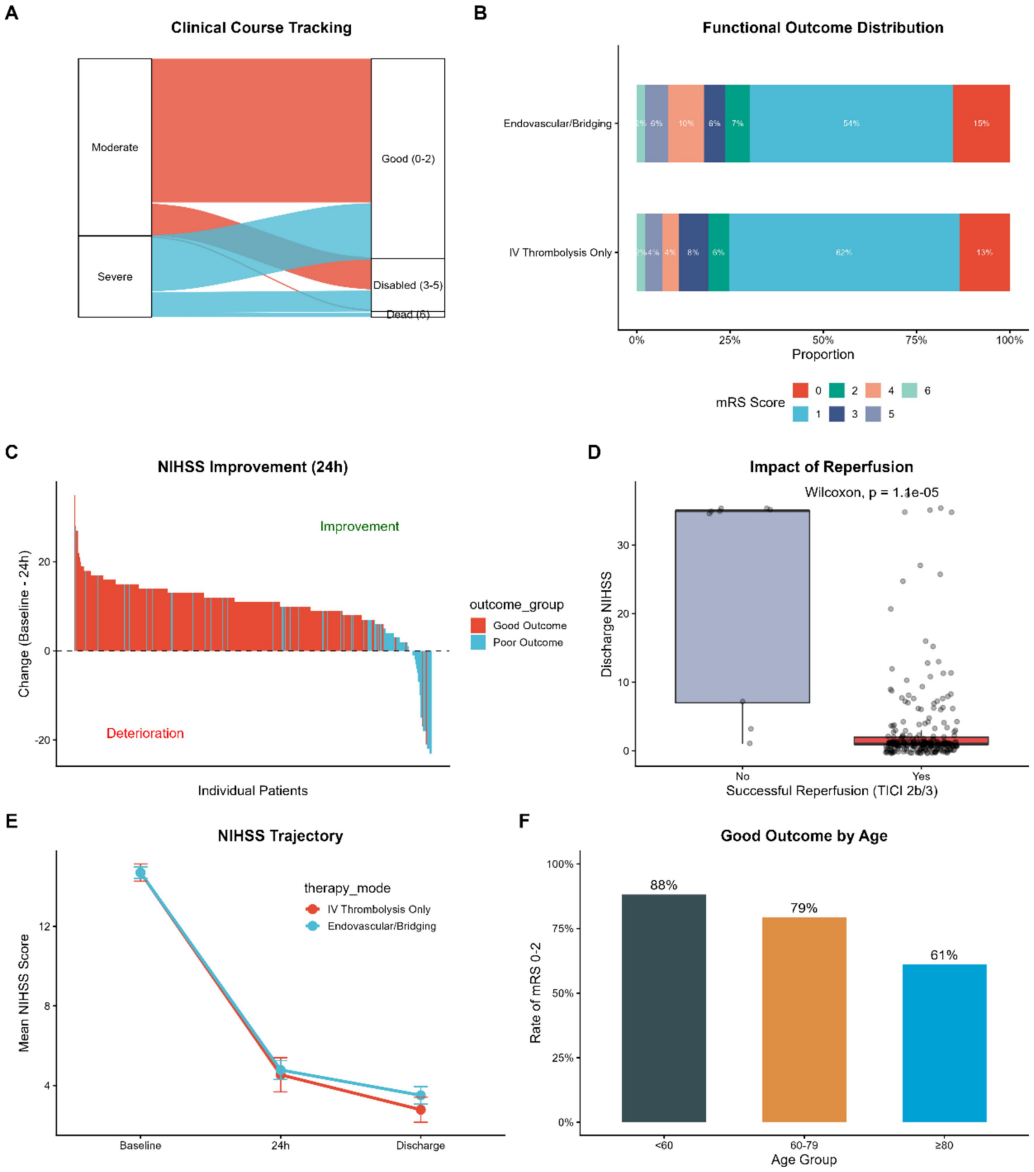
Clinical efficacy outcomes in the entire cohort. **(A)** Sankey diagram visualizing the clinical course from admission severity (NIHSS categories) to discharge functional outcome (mRS categories). **(B)** Grotta bars showing the distribution of mRS scores at discharge stratified by therapy mode. **(C)** Waterfall plot of individual changes in NIHSS score from baseline to 24 h; bars above zero indicate improvement. **(D)** Boxplot showing the impact of successful reperfusion (mTICI 2b/3) on discharge NIHSS scores. **(E)** Trajectory of mean NIHSS scores from baseline to discharge. **(F)** Rates of good functional outcome (mRS 0–2) stratified by age group. NIHSS, National Institutes of Health Stroke Scale; mRS, modified Rankin Scale.

The therapeutic efficacy was first evaluated through the longitudinal assessment of neurological severity. As shown in Figure 5E, mean NIHSS score demonstrated a progressive reduction from baseline to 24 h with a further decrease by discharge showing a long-term neurological outcome in the general cohort. This positive clinical trajectory is visually reconstructed in the Sankey diagram (Figure 5A), where a substantial proportion of patients with severe neurological impairment at the onset of treatment transitioned into categories of favorable functional outcome (Good Outcome) by the time of discharge.

As to the primary endpoint, the rate of functional independence (mRS 0–2) was comparable between the Tenecteplase (38.2%) and Alteplase (40.3%) groups, and they did not show any significant difference when adjusted by potential confounders (aOR 0.91, 95% CI 0.52 1.58; Table 2). This observation was further enhanced by the distribution of mRS scores in the entire spectrum of disability (Figure 5B, Grotta bars), showing similar patterns of disability shifts between the two treatment modalities. At the individual level, the waterfall plot (Figure 5C) illustrates the heterogeneity of early treatment response; most of the patients developed or showed a positive change in NIHSS scores at 24 h (bars above zero) which is an early neurological improvement (ENI).

The pivotal role of successful vessel recanalization in driving these favorable outcomes was underscored in Figure 5D. The median discharge NIHSS scores of patients with successful reperfusion (mTICI 2b/3) were significantly lower than in those with ineffective reperfusion (*p* < 0.001) confirming that angiographic success remains the fundamental prerequisite for neurological recovery in large vessel occlusion strokes due to large vessel occlusion.

Lastly, we examined demographic prognosis determinants. As shown in Figure 5F, age remained a potent predictor of functional recovery. The rate of good outcomes showed an inverse relationship with age, peaking at 88% in patients under 60 years and decreased with age to 61-percent in patients at the age of 80 (or older) years. This is an indication that as effective as reperfusion therapy is, the physiological reserve associated with age continues to influence the ceiling of clinical recovery.

### 3.6 Comparative outcomes between Tenecteplase and Alteplase after propensity score matching (PSM)

**Figure 6:**
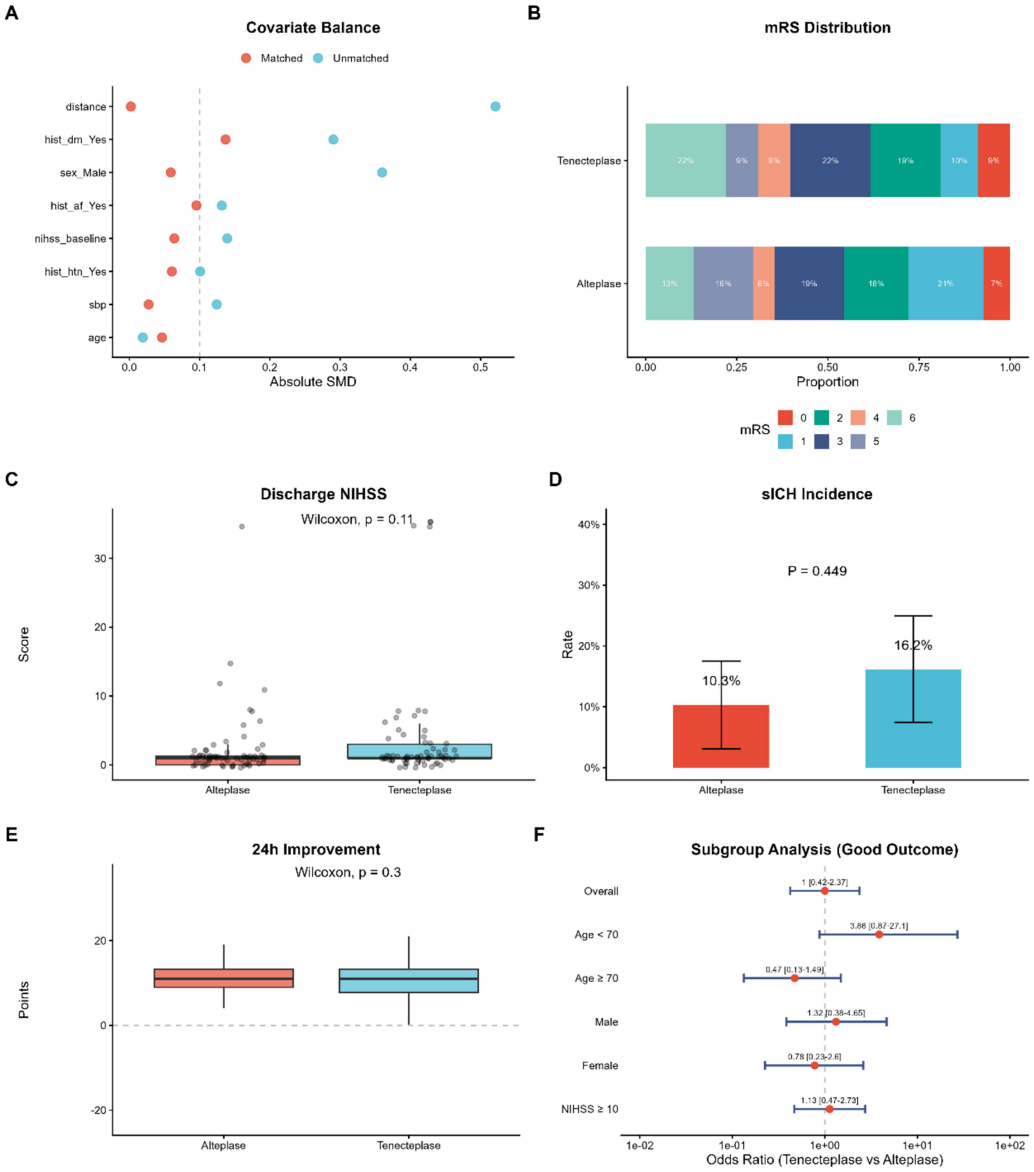
Comparative outcomes between Tenecteplase and Alteplase after propensity score matching (PSM). **(A)** Love plot displaying the absolute standardized mean differences (SMD) of covariates before and after matching; all SMDs were <0.1 after matching. **(B)** Distribution of mRS scores in the matched cohorts. **(C)** Comparison of discharge NIHSS scores between the matched groups. **(D)** Incidence of sICH in the matched cohorts (*p* > 0.05). **(E)** Comparison of 24-h NIHSS improvement. **(F)** Subgroup analysis (forest plot) showing the odds ratios (OR) for good functional outcome (Tenecteplase vs. Alteplase) across key clinical subgroups. PSM, propensity score matching; SMD, standardized mean difference; OR, odds ratio.

To rigorously adjust for the baseline disparities identified in the unmatched cohort—particularly the imbalances in sex and stroke history—we performed a 1:1 propensity score matching (PSM) analysis. The effectiveness of this procedure is visualized in the Love plot (Figure 6A) that shows that there is a significant decrease in covariate bias. The absolute standardized mean differences (SMD) for all included variables were minimized post-matching and would imply that the two treatment drugs were well balanced and matched.

In the matched cohort, the therapeutic efficacy of Tenecteplase mirrored that of Alteplase. The distribution of functional outcomes on both sides of the spectrum, which was found by the distribution of functional outcomes on the modified Rankin Scale (Figure 6B), revealed a similar pattern of disability with no statistically significant shift in the proportion of the patients with functional independence. The quantitative measurement of the neurological severity also supported this result; the median NIHSS scores at discharge were also similar in both the Tenecteplase and Alteplase (*p* = 0.11, Figure 6C). Moreover, in terms of the early recovery dynamicals, the magnitude of NIHSS improvement at 24 h did not differ significantly between the two agents (*p* = 0.3, Figure 6E) suggesting that both thrombolytics facilitate a similar trajectory of early neurological restoration.

Safety outcomes in the matched cohort were consistent with the findings from the multivariable regression analysis. As shown in Figure 6D, the incidence of symptomatic intracranial hemorrhage (sICH) was 16.2% in the Tenecteplase and Alteplase, respectively. Although the rate was numerically higher in the Tenecteplase arm, this difference did not reach statistical significance (*p* = 0.449), suggesting that the hemorrhagic risk profile of Tenecteplase is not statistically inferior to that of Alteplase in this real-world setting.

Lastly, Figure 6F utilized the subgroup analysis to understand whether the treatment effects were consistent across the important clinical strata. The general odds ratio to have a good functional outcome was 1.00 (95% CI 0.42–2.37), and this indicating equipoise between the two treatments. While a trend favoring Tenecteplase was observed in younger patients when adjusted for older (Age < 70) and younger (Age ≥ 70) patients (OR 3.86 and OR 0.47 respectively), the wide confidence intervals and the coverage of unity of most subgroups make it impossible to make final conclusions about the best efficacy in this or that subgroup. All these findings support the conclusion that Tenecteplase is not inferior to Alteplase in a wide spectrum of clinical manifestations.

## 4 Discussion

While intravenous thrombolysis remains the cornerstone of acute ischemic stroke management, the real-world comparative efficacy of Tenecteplase versus Alteplase, particularly in unselected cohorts with heavy disease burdens, warrants further validation (17). This single-center retrospective study demonstrates that Tenecteplase offers non-inferior functional outcomes and a comparable safety profile to Alteplase under a non-randomized real-life work environment, which supports its use as a valid therapy alternative in the everyday clinical practice.

In terms of clinical efficacy, our results are consistent with those of the EXTEND-IA TNK and AcT trials that determined the non-inferiority of Tenecteplase (17). The rate of functional independence (mRS 0–2) observed in our study (approximately 40%) was, nevertheless, lower in numerical terms than the results of 50–60 percent of those randomized controlled trials. Such a difference can probably be explained by the unique features of our population of the real-world, which presented with a higher median baseline NIHSS score (14 vs. 10–12 in trials) and had a more advanced age distribution (18). Figure 6F (our subgroup analysis) also revealed that although there was a good trend in favor of prior studies in younger patients, suggest that physiological reserve and collateral status may play a more decisive role than the choice of lytic agent in determining outcomes for critically ill patients (19).

Regarding safety, particularly symptomatic intracranial hemorrhage (sICH), our propensity score-matched analysis (Figure 6D) revealed a numerically higher rate in the Tenecteplase group compared to the Alteplase group (16.2% vs. 10.3%). Although this difference was not statistically significant, we cannot definitively claim safety equivalence due to the limited sample size (*n* = 68 for Tenecteplase) and the resulting wide confidence intervals. It is highly noteworthy that the overall sICH rate in our matched cohort noticeably exceeds the 3 to 7% typically reported in major RCTs (20). We hypothesize that this elevated sICH rate is a manifestation of real-world heterogeneity and inherent selection bias. Specifically, our unselected cohort presented with a high baseline stroke severity (median NIHSS of 14) and a high proportion of large vessel occlusions requiring endovascular thrombectomy, both of which are established risk factors for hemorrhagic transformation. As demonstrated in our multivariable analysis (Figure 4E), higher baseline NIHSS and admission systolic blood pressure emerged as independent predictors of hemorrhage (21). Consequently, while a non-inferiority trend in safety is observed, the high sICH rate underscores the need for cautious patient selection in routine clinical practice rather than implying absolute safety equivalence (21, 22).

Several limitations of this study must be acknowledged. First, the retrospective, single-center design is inherently susceptible to selection bias. Even though propensity score matching was utilized to balance observable covariates, the presence of unmeasured confounders (e.g., frailty index, precise collateral scores) could still affect the findings. Second, the Tenecteplase group sample size was remarkably small (*n* = 68). This severely limits the statistical power to definitively identify small differences in safety endpoints and resulted in wide confidence intervals in the multivariable and subgroup analyses, meaning that potential safety concerns cannot be entirely ruled out. Third, being a real-world study, the decision-making process for selecting between bridging therapy and direct thrombectomy, as well as the choice of lytic agent, was heterogeneous and dependent on operator preference and drug availability, which further introduces selection bias and may influence the generalizability of the workflow metrics. Finally, and importantly, our study did not possess a sufficient sample size to analyze highly clinically relevant off-label scenarios that are uniquely valuable in real-world data, such as thrombolysis beyond 4.5 h or use in patients receiving oral anticoagulants. Furthermore, detailed procedural timelines beyond door-to-needle time, such as imaging-to-needle time, were not systematically recorded. The absence of these analyses limits the conceptual advancement and translational relevance of our findings regarding the optimization of in-hospital treatment efficiency. In conclusion, this real-world study provides evidence suggesting a non-inferiority trend of Tenecteplase compared to Alteplase regarding functional independence and successful reperfusion in the treatment of acute ischemic stroke, without compromising workflow efficiency. While the incidence of symptomatic intracranial hemorrhage was numerically higher in the Tenecteplase group, the wide confidence intervals prevent a definitive conclusion of safety equivalence. The overall high bleeding rate highlights the complexities of treating a real-world cohort with severe neurological deficits. These findings support Tenecteplase as a viable therapeutic alternative in daily practice, but underscore that future large-scale, adequately powered multicenter registries are strictly warranted to validate its definitive safety profile and refine patient selection criteria.

## 5 Conclusion

In conclusion, this real-world study provides evidence supporting the non-inferiority of Tenecteplase compared to Alteplase in the treatment of acute ischemic stroke, including a significant proportion of patients with large vessel occlusions. Tenecteplase achieved comparable rates of functional independence and successful reperfusion without compromising workflow efficiency, as evidenced by similar door-to-needle times. While the incidence of symptomatic intracranial hemorrhage was numerically higher in the Tenecteplase group, the difference was not statistically significant and appears attributable to the high baseline severity of the cohort rather than drug-specific toxicity. These findings reinforce the clinical utility of Tenecteplase as a practical and effective therapeutic alternative in daily practice. Future large-scale, multicenter registries are warranted to validate these observations and further optimize patient selection criteria for specific thrombolytic agents.

## Data Availability

The raw data supporting the conclusions of this article will be made available by the authors, without undue reservation.
